# Acute Ischemic Stroke in an Infant With COVID-19 Infection and Delayed Diagnosis of Neurofibromatosis Type 1

**DOI:** 10.7759/cureus.51524

**Published:** 2024-01-02

**Authors:** Asra Akbar, Sean Creeden

**Affiliations:** 1 Pediatric Neurology, University of Texas Health Science Center at San Antonio, San Antonio, USA; 2 Pediatric Neurology, University of Illinois College of Medicine Peoria, Peoria, USA; 3 Radiology, University of Illinois College of Medicine Peoria, Peoria, USA

**Keywords:** neurofibromatosis type 1, covid-19, sars-cov-2 (severe acute respiratory syndrome coronavirus-2), cafe-au-lait spots, neurofibromatosis, pediatric stroke

## Abstract

Acute ischemic stroke is an uncommon presentation in the pediatric population as compared to the elderly population. COVID-19 infection is associated with several neurological manifestations, with ischemic strokes being underrecognized. Cerebrovascular events associated with COVID-19 may be due to systemic inflammation and hypercoagulable state.

Neurofibromatosis type 1 (NF1) is an inherited multisystem disorder caused by dominant loss-of-function mutations of the tumor-suppressor gene neurofibromin 1, which is located at 17q11.2.1. NF1 is associated with multiple cerebrovascular abnormalities, including internal carotid artery occlusion.

A review of the current literature on manifestations of COVID-19 in the pediatric population, including stroke and seizures, is also provided in this case report. A brief review of the literature on neurofibromatosis and the risk of stroke as well as other clinical manifestations is also included as a part of this case report.

This case illustrates the importance of recognizing acute and rare complications of neurofibromatosis. Cerebral vasculopathy is an important but underrecognized complication of NF1. Children with neurofibromatosis and hypertension require a thorough and complete neurologic evaluation.

This case describes a young infant with a delayed clinical diagnosis of NF1 who was presented with viral manifestations of COVID-19 infection and was diagnosed with a large middle cerebral artery stroke.

## Introduction

Coronavirus disease 2019 (COVID-19) is caused by severe acute respiratory syndrome coronavirus 2 (SARS-CoV-2). Even though the most common presentation is upper respiratory tract symptoms, other wide varieties of multi-system and neurological manifestations have been reported [1]. Ischemic stroke, specifically in the pediatric population, is a less commonly reported presentation. SARS-CoV-2 is reported to cause a cytokine storm through the activation of angiotensin-converting enzyme receptor binding, which then leads to a hypercoagulable state and thus an increased incidence of thrombosis in patients with COVID-19 infection, specifically at higher risk of stroke and other neurological manifestations due to chronic conditions, such as neurofibromatosis type 1 (NF1) [2].

Although the association of strokes and von Recklinghausen neurofibromatosis (NF1) in young children is less commonly reported, it is an important complication of this disorder.

Hornstein et al. [3] reported a case of a seven-week-old infant with NF1 who presented with an ischemic stroke prior to the clinical manifestation of neurofibromatosis.

Cerebral vasculopathy is an important but underrecognized complication of NF1 [4].

## Case presentation

A six-month-old infant presented with lethargy in June 2022. A few hours later, he had a short episode of eye blinking and eye flutter. The mother brought the patient to the hospital where it was noted that there was some right-sided twitching and fussiness, and there was gaze deviation to the right side. The exam was significant for right-sided hemiparesis. Seven cafe-au-lait spots were seen ranging between 1 and 6 mm in size.

The infant had four cafe-au-lait spots at birth, but the diagnosis was not made until this admission as the size and number of cafe-au-lait spots had increased by the age of six months.

**Figure 1:**
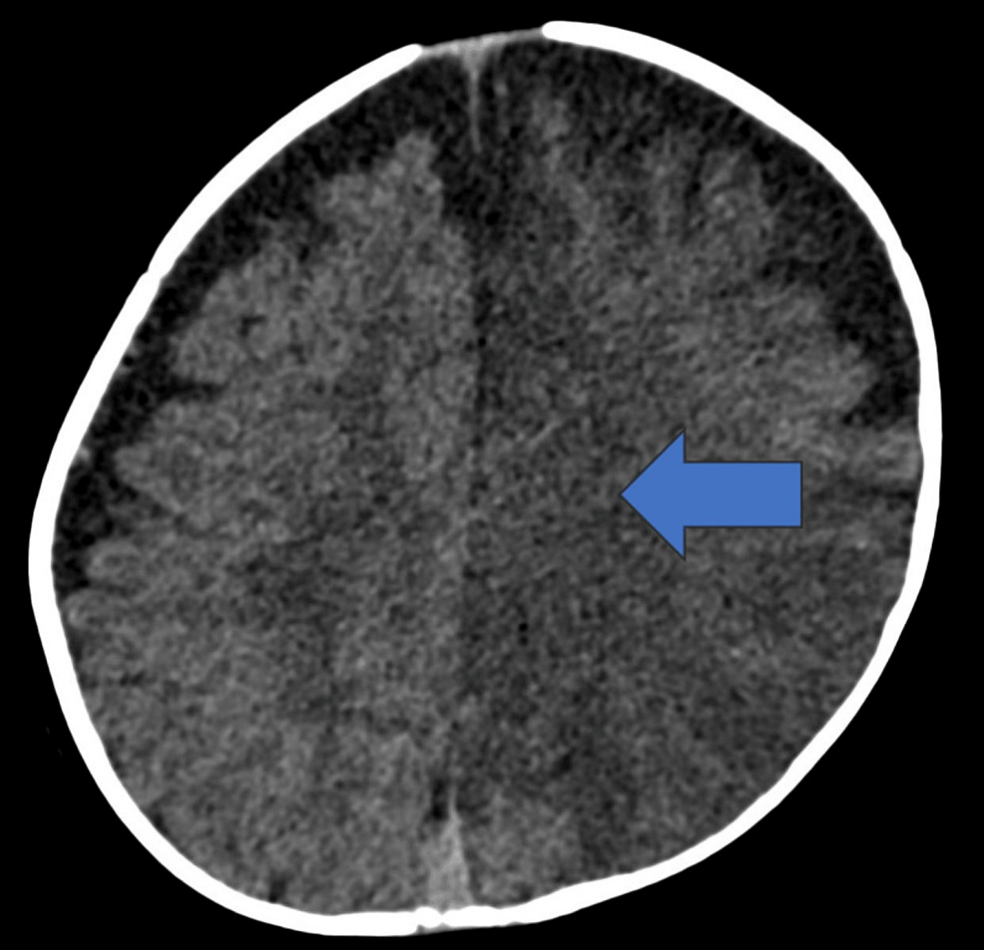
Axial non-contrast CT of the head demonstrates a large area of hypoattenuation and loss of gray-white matter differentiation in the left cerebral hemisphere. The blue arrow shows a large area of hypoattenuation and loss of gray-white matter differentiation in the left cerebral hemisphere.

The CT scan of the head showed a large left middle cerebral artery (MCA) distribution infarct but there was some suggestion that the infarct was secondary to cerebral sinus venous thrombosis (CSVT) (Figure 1). In the meantime, the infant was tested positive for COVID-19. The patient was not a candidate for tissue plasminogen activator (tPA) because of the high risk of intracranial hemorrhage and age. At the suggestion of hematology, he was started on unfractionated heparin. Hematology was also asked to consult for management recommendations from the point review of evaluation for the prothrombotic state.

CT angiography (CTA) was done to clarify the questions and showed high-grade stenosis or subocclusive thrombus involving the mid-distal M1 segment of the left MCA. Neck CTA was normal. Intracranial CT showed no evidence of dural venous sinus thrombosis; however, the left transverse and sigmoid sinus were relatively hypoplastic.

**Figure 2:**
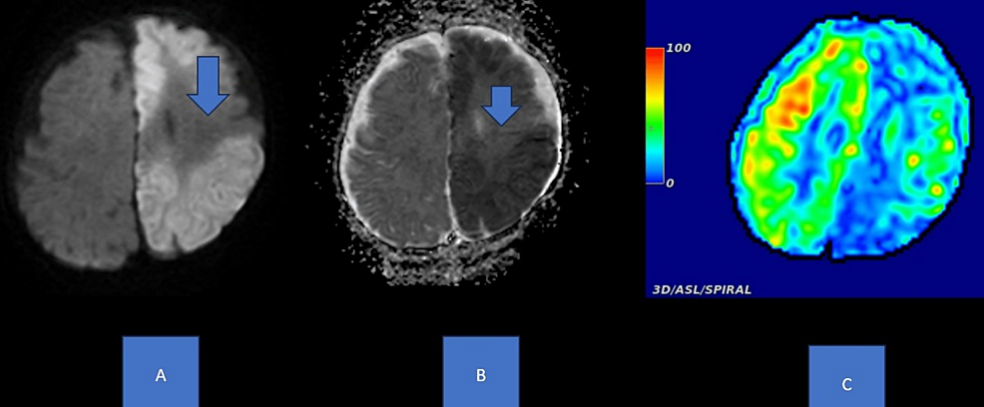
Axial DWI, ADC, and ASL demonstrate a large area of restricted diffusion and decreased cerebral blood flow in the left cerebral hemisphere with some sparing of the left frontal lobe. DWI: diffusion-weighted imaging; ADC: apparent diffusion coefficient; ASL: arterial spin labeling.

**Figure 3:**
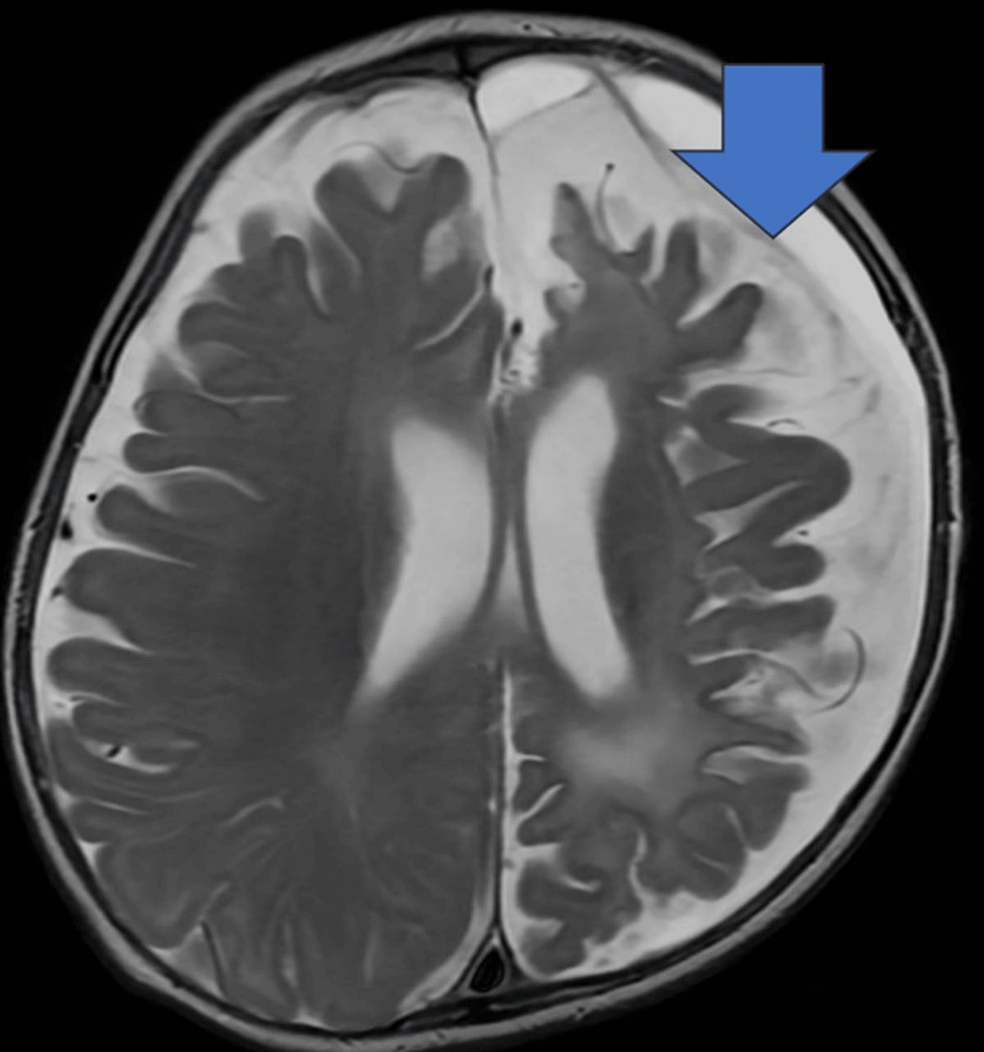
Axial T2 image on follow-up MRI of the brain several months later demonstrates encephalomalacia, gliosis, and volume loss in the left cerebral hemisphere with the development of a likely compensatory subdural hygroma along the left cerebral convexity (Monro-Kellie doctrine). The blue arrow indicated encephalomalacia, gliosis, and volume loss in the left cerebral hemisphere with the development of a likely compensatory subdural hygroma along the left cerebral convexity.

MRI of the brain was also done, which showed an acute ischemic infarct involving the left cerebral hemisphere for mild local mass effect and sparing the left posterior cerebral artery (PCA) territory (Figures 2, 3).

This semiology of seizures includes eye blinking often and eye deviation to the right. EEG showed left posterior focal discharges but no clear electrographic events. The patient was initially started on 40 milligrams/kilogram of Keppra. The events improved but continued and the total dosage was increased to 60 milligrams/kilogram of Keppra by giving an additional 20 milligrams/kilogram. That did not help so phenobarbital at 20 milligrams/kilogram was started, which stopped any clear clinical or electrographic episodes. The seizures ceased.

No further seizures and or seizure-like spells have been reported since discharge from the hospital. He did not had seizures prior to that admission and stroke.

## Discussion

Acute strokes are uncommonly encountered in the pediatric population with an incidence of 1.2 to 2.1 out of 100,000 [5]. During the COVID-19 pandemic, neurological complications, such as acute ischemic stroke, in the pediatric population were recognized even though there are isolated cases of acute ischemic stroke with COVID-19 in the pediatric age group [6].

COVID-19 caused by SARS-CoV-2 was first described in December 2019. Anosmia, cranial nerve deficits, meningitis, seizures, and stroke are neurological complications of COVID-19 infection [7]. Neurological manifestations of COVID-19 with CNS symptoms are reported in 25% of patients [8].

NF1 is an inherited multisystem disorder caused by dominant loss-of-function mutations of the tumor-suppressor gene neurofibromin 1, which is located at 17q11.2.1. Cerebral vasculopathy in NF1 can be missed and remain silent for a prolonged duration of time. Thus, the pediatrician's role is critical for the timely diagnosis of neurofibromatosis [9]. The diagnosis of NF1 is made if the patient meets two of the following criteria, including the presence of cafe-au-lait spots, intertriginous freckling, dermal neurofibroma, optic glioma, Lisch nodules, skeletal dysplasia, and positive family history [10].

NF1 is associated with multiple cerebrovascular abnormalities, including internal carotid artery (ICA) stenosis/occlusion [11].

There have been previous case reports regarding children developing stroke following COVID-19 infection.

Kihira et al. in June 2020 reported an unfortunate case of a healthy five-year-old child who presented with respiratory symptoms of COVID-19 infection complicated by cardiogenic shock and hypercoagulable state, which presumably led to a massive cerebral infarction with devastating manifestation [12].

Morgan et al. [6] in July 2022 reported a case of a two-year-old child who was diagnosed with COVID-19 infection and developed multisystem inflammatory syndrome and large vessel ischemic stroke, which required decompressive craniotomy. He improved and was discharged with moderate disability.

Kanğın et al. [13] reported a case of a seven-year-old previously healthy child with COVID-19 infection and catastrophic intracranial arterial thromboembolic event leading to brain death.

Appavu et al. [5] reported two previously healthy children aged eight years old and 16 years old, who suffered disabling arterial ischemic strokes because of acute intracranial large vessel occlusion within three to four weeks of COVID-19. Neither of the children had symptoms of multisystem inflammatory syndrome and fever.

AlKandari et al. [14] reported a case of a previously healthy 15-year-old with COVID-19 infection and acute ischemic stroke with a return to baseline.

This case illustrates the importance of recognizing both these complications of neurofibromatosis. Children with neurofibromatosis and hypertension require careful neurologic evaluation.

There is limited data about the underlying mechanisms of neurological disorders caused by COVID-19. An estimated 2% of patients have neurological symptoms, and even limited vision is available in the pediatric population. Some other pathophysiologies of ischemic stroke in COVID-19 are cerebral hypoperfusion, hypertension, and septic embolization [6]. Again, this case highlights an important point about underlying chronic diseases such as NF1 in the pediatric population, which predisposes the patient to similar complications.

## Conclusions

There is a paucity of clinical data in the pediatric population about neurological cases associated with COVID-19 infection, thus physicians must develop a better understanding of the infection in more vulnerable populations at a higher risk of stroke or epilepsy. The diagnosis of neurofibromatosis also puts the patient at a greater risk for the development of stroke, which could have been exacerbated by the COVID-19 infection.
